# Catechol End-Functionalized Polylactide by Organocatalyzed Ring-Opening Polymerization

**DOI:** 10.3390/polym10020155

**Published:** 2018-02-06

**Authors:** Naroa Sadaba, Maitane Salsamendi, Nerea Casado, Ester Zuza, Jone Muñoz, Jose-Ramon Sarasua, David Mecerreyes, Daniele Mantione, Christophe Detrembleur, Haritz Sardon

**Affiliations:** 1 POLYMAT, University of the Basque Country UPV/EHU, Joxe Maria Korta Center, Avenida Tolosa 72, 20018 Donostia/SanSebastian, Spain; naroa.sadaba@polymat.eu (N.S.); maitane.salsamendi@ehu.es (M.S.); nerea_casado001@ehu.es (N.C.); david.mecerreyes@ehu.es (D.M.); daniele.mantione@ehu.es (D.M.); 2 Department of Mining-Metallurgy Engineering and Materials Science, POLYMAT, University of the Basque Country UPV/EHU, School of Engineering, Alameda de Urquijo s/n, 48013 Bilbao, Spain; ester.zuza@ehu.es (E.Z.); jone.munoz@ehu.es (J.M.); jr.sarasua@ehu.eus (J.-R.S.); 3 IKERBASQUE Basque Foundation for Science, Maria Diaz de Haro 3, E-48011 Bilbao, Spain; 4 Center for Education and Research on Macromolecules (CERM), CESAM Research Unit, University of Liège (ULg), Sart Tilman B6a, Liège, Belgium; christophe.detrembleur@ulg.ac.be

**Keywords:** ring opening polymerization, dopamine, catechol, quinone, polylactide

## Abstract

There is a great interest in incorporating catechol moieties into polymers in a controlled manner due to their interesting properties, such as the promotion of adhesion, redox activity or bioactivity. One possibility is to incorporate the catechol as end-group in a polymer chain using a functional initiator by means of controlled polymerization strategies. Nevertheless, the instability of catechol moieties under oxygen and basic pH requires tedious protection and deprotection steps to perform the polymerization in a controlled fashion. In the present work, we explore the organocatalyzed synthesis of catechol end-functional, semi-telechelic polylactide (PLLA) using non-protected dopamine, catechol molecule containing a primary amine, as initiator. NMR and SEC-IR results showed that in the presence of a weak organic base such as triethylamine, the ring-opening polymerization (ROP) of lactide takes place in a controlled manner without need of protecting the cathechol units. To further confirm the end-group fidelity the catechol containing PLLA was characterized by Cyclic Voltammetry and MALDI-TOF confirming the absence of side reaction during the polymerization. In order to exploit the potential of catechol moieties, catechol end-group of PLLA was oxidized to quinone and further reacted with aliphatic amines. In addition, we also confirmed the ability of catechol functionalized PLLA to reduce metal ions to metal nanoparticles to obtain well distributed silver nanoparticles. It is expected that this new route of preparing catechol-PLLA polymers without protection will increase the accessibility of catechol containing biodegradable polymers by ROP.

## 1. Introduction

Catechol-derivatives and their corresponding ortho-quinones represent one of the most important classes of naturally abundant small molecules due to their capacity to mediate fascinating chemical reactions. Indeed, catechol molecules not only have demonstrated to be potent antioxidant, anti-inflammatory, redox active, or to present outstanding adhesion to different substrates but also they could be easily oxidized. When oxidized, this compound possesses the capacity to react towards a wide variety of chemical groups such as amines or thiols. It is not surprising, that these amazing properties of catechols have opened the door to the design of new (multi)functional polymers based on catechols. Catechols are used in several fields, such as tissue engineering, due to biocompatible and non-toxic properties, for surface coating to protect or improve the adhesion of a given material to a substrate [1,2,3,4,5], as redox-active polymers (RAPs) in the battery field to prepare robust energy storage devices [6]. Catechol units can serve also as reducing agent for the preparation of well-dispersed silver nanoparticles (AgNPs) leading to hydrogels with antibacterial behavior [7].

Catechol functionality can be incorporated into polymers using different strategies. The most studied strategy is the direct polymerization of functional catechols such as dopamine either by oxidative [5] or enzymatic [8] routes. Catechol units can be easily oxidized into highly reactive quinones promoting the auto-polymerization of catechol units and the formation of tridimensional structures. More recently, in order to obtain well-defined linear polymers with pending catechol unit, the free-radical polymerization of vinylic monomers bearing catechol has also been investigated. Although catechols are well-known polymerization inhibitors, their polymerization with no catechol protection has reported the viability of this approach. However, in most of these studies, even using controlled radical polymerization techniques, the potential side reactions of propagating radicals with catechols and their effect on the polymer architecture were rarely discussed. As shown by Detrembleur et al., when vinyl monomers bearing catechols were not protected, high molar mass (hyper)branched polymers were formed at low catechol content [9,10]. At high contents, a crosslinked material was obtained. This has later been further confirmed by Kamperman et al. [11,12].

Besides free radical polymerization, catechol units have been also investigated as interesting synthetic targets in ring-opening polymerization (ROP) reactions. Thus, Deming et al. prepared high molecular weight polypeptides bearing catechols by ROP of α-amino acid *N*-carboxyanhydrides NCAs containing protected catechols followed by their deprotection [13]. Although, catechol units did not interfere with nucleophilic substitution reactions, their instability under oxygen and basic pH represent difficulties to perform controlled ROP [9]. Thus, it is recommended to protect and unprotect catechol units during the polymerization process in order to avoid its homopolymerization and to carry out a controlled polymerization [14,15,16].

In the last decade, continuous efforts have been devoted to promote innovations in polymerization towards the convergence of functional group tolerance, fast rates, and selectivity in catalyst design. For instance, Waymouth et al. recently developed an effective catalytic system combining alkoxides with thioureas that catalysed rapid and selective ring-opening polymerizations, while most of the catalysts were subject to find a compromise between polymerization rate and polymerization control [17]. Following this innovation, in this paper, we use for the first time an unprotected catechol containing a pendant amine group (named dopamine) as initiator for the controlled ROP of l-lactide (l-LA) using a weak base as catalyst. We performed the polymerizations in the absence of oxygen and using different solvents in order to reduce the potential of catechol units to autopolymerize [11,18,19]. The success of the polymerization is evaluated by Hydrogen and Carbon Nuclear Magnetic Resonance (^1^H and ^13^C NMR), size exclusion chromatography (SEC) and MALDI-TOF. To the best of our knowledge, this is the first example of the utilization of unprotected catechol initiators for ROP of l-lactide carried out without suffering any side reaction.

## 2. Materials and Methods

### 2.1. Materials

l-lactide (l-LA) was obtained from Futerro (Escanaffles, Belgium), and it was recrystallized and dried prior to use. Triethylamine (TEA), dopamine hydrochloride and benzoic acid were purchased from Sigma Aldrich (St. Louis, MO, USA) and were dried under vacuum for 24 h. Dichloromethane (DCM) (≥99%) and tetrahydrofuran (THF) (≥99%), chloroform, 99% extra dry and methanol were attained from Fisher (Madrid, Spain) and used as received. *N*,*N*-Dimethylformamide (DMF) was acquired from SeccoSolv (St. Louis, MO, USA).

### 2.2. Measurements

Nuclear Magnetic Resonance (NMR), Size exclusion chromatography (SEC) and MALDI-TOF Mass Spectrometry measurements were used for determine the polymers characteristics. ^1^H and ^13^C NMR spectra were recorded at room temperature on Bruker spectrometers (Bruker, Billerica, MA, USA) operating at 300 MHz, using deuterated chloroform (CDCl_3_) as solvent. The molecular weights of the samples were determine by SEC. SEC was performed in THF at 30 °C using a Waters chromatograph (waters chromatography, Milford, MA, USA) equipped with four 5 mm Waters columns (300 mm × 7.7 mm) connected in series with increasing pore sizes (100, 1000, 105, 106 Å). Toluene was used as a marker. Polystyrene of different molecular weights, ranging from 2100 g·mol^−1^ to 1,920,000 g·mol^−1^, were used for the SEC calibration. MALDI-TOF Mass Spectrometry measurements were performed on a Bruker Autoflex Speed system (Bruker, Billerica, MA, USA) instrument equipped with a 355 nm Nd:YAG laser. All spectra were acquired in the positive-ion reflection mode (accelerating voltage 20 kV, pressure 5 × 10^−6^ mbar). Samples were dissolved at concentration of 10 g·L^−1^ in THF.

UV-Vis spectroscopy and Cyclic Voltammetry were used to show the oxidation-reduction of the polymers. UV-Vis spectroscopy was performed on a Shimadzu UV-2550 spectrophotometer using 1 cm path length quartz cells. The polymer Catechol-PLLA acted as both the reducing agent and the stabilizer of AgNPs. The polymer solution with a 0.25 mM effective dopamine concentration was obtained by dissolving Catechol-PLLA (2.8 mg) in 3 mL of THF. AgNO_3_ (0.5 mM, 0.25 mg, 0.0015 mmol of Ag^+^) dissolved in 100 µL of dimethyl sulfoxide (DMSO) was then added to the polymer solution and the mixture stirred for 13 h at 25 °C. Extinction spectroscopy and transmission electron microscopy (TEM), were used to study the formation of the AgNPs over time (PLLA dopamine-Ag dispersions were obtained). In the case of Cyclic Voltammetry, electrochemical measurements were performed on an Autolab PGSTAT302N potentiostat (Metrohm Autolab, Utrecht, The Netherlands) using the standard three-electrode cell with a glassy carbon electrode (GC, 0.07 cm^2^ area) and platinum plate as working (WE) and counter (CE) electrodes, respectively and an Ag/AgCl (3 M KCl) reference electrode. The PLLA-catechol electrodes were drop-casted on GC electrode from a dichloromethane solution. Cyclic voltammetry (CV) was performed from −0.25 V to 1.0 V vs. Ag/AgCl at various scan rates in 0.1 M perchloric acid aqueous solution (HClO_4_, Sigma-Aldrich, St. Louis, MO, USA).

### 2.3. Synthesis of l-Lactide

#### 2.3.1. Ring-Opening Polymerization of l-Lactide Using Dopamine as Initiator

All reactions were carried out in a nitrogen-purged glovebox. Dopamine hydrochloride (1 equiv.) was dissolved in DMF (100 M). Then a solution of CHCl_3_ (2 M respect to the monomer) containing TEA (1.25 equiv.) and l-lactide (10, 25 or 50 equiv.) was added to the dopamine solution. The solution was stirred at room temperature for 24 h (^1^H NMR monitoring). The reaction catalyst was quenched with an excess of benzoic acid and the polymer solution was precipitated into cold methanol, centrifuged and dried to obtain a white solid (yield 80%). ^1^H NMR (300 MHz, Chloroform-d) δ 6.89–6.55 (m, 2H), 6.27 (t, *J* = 5.7 Hz, 1H), 5.20 (ddt, *J* = 8.8, 7.9, 7.0 Hz, 20H), 4.38 (q, *J* = 6.9 Hz, 1H), 3.49 (d, *J* = 6.0 Hz, 1H), 2.73 (s, 1H), 1.66–1.54 (m, 62H).

#### 2.3.2. Ring-Opening Polymerization of l-Lactide Using Phenethylamine as Initiator

The reaction was carried out in a nitrogen-purged glovebox. Phenethylamine was dissolved in DMF (100 M), then a solution of CHCl_3_ (2 M) containing l-lactide (50 equiv.) and TEA (1.25 equiv.) was mixed with phenethylamine solution. Finally, the reaction was kept during 24 h. The reaction was precipitated into cold methanol, centrifuged and dried to obtain a white solid (yield 83%).

#### 2.3.3. Synthesis of Silver Nanoparticles (AgNPs)

The synthesis of AgNPs was carried out by the reduction of Ag^+^ to Ag^0^. The polymer Catechol-PLLA (DP = 50) acted as both the reducing agent and the stabilizer of AgNPs. The polymer solution with a 0.25 mM effective dopamine concentration was obtained by dissolving Catechol-PLLA (2.8 mg) in 3 mL of THF. AgNO_3_ (0.5 mM, 0.25 mg, 0.0015 mmol of Ag^+^) dissolved in 100 µL of DMSO was then added to the polymer solution and the mixture stirred for 13 h at 25 °C. Extinction spectroscopy and transmission electron microscopy (TEM), were used to study the formation of the AgNPs over time (PLLA dopamine-Ag dispersions were obtained).

## 3. Results and Discussion

### 3.1. Polymerization and Characterization of Catechol-PLLA

**Scheme 1:**
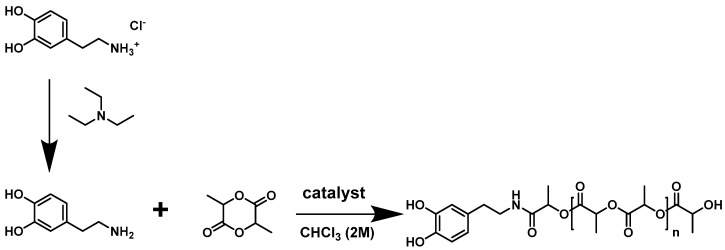
Ring-opening polymerization for dopamine end-capped polylactide (PLLA).

As a first test, we investigated the catalyst free ROP of l-LA (Table 1, entry 1) in the presence of dopamine initiator (Scheme 1). In order to prepare the free amine and allow the initiation process, 1.0 equiv. of triethylamine (TEA) must be added to a solution of dopamine hydrochloride (1 equiv.) followed by the addition of l-lactide monomer and chloroform. Although amines can act as initiator, the reaction did not lead to any polymerization even using different solvents such as chloroform, THF or DMF and temperatures (Supplementary Materials Table S1).

After this unsuccessful attempt, we envisioned a more efficient ROP using an organic base inspired by the work of Bourissou and co-workers which explored 1,8-diazabicyclo[5.4.0]undec-7-ene (DBU) catalyst for the use of amines as initiators for the preparation of amide end-capped PLLA [14]. Two different bases were explored as potential catalyst for the catechol initiated controlled polymerization of l-LA, triethylamine (TEA) a weak base and DBU a strong base (Table 1, entry 2 and Supplementary Materials Figure S2, respectively). For catalyst screening, polymerization of l-LA was performed at room temperature in chloroform using a 2 M concentration of l-LA and an initial monomer-to-initiator-to-catalyst ratio of ([M]_0_/[I]_0_/[catalyst]) of 10/1/0.25). The polymerization was monitored by ^1^H NMR and the conversion was calculated by determining the ratio of the signals related to the monomer methine protons at 5.4 ppm from and l-LA, and comparing them to the signal of the polymer methine protons at 5.2 ppm from poly(l-lactide) (Supplementary Materials Figure S1).

We found that in the presence of 0.25 equiv. of DBU polymer was formed but the molecular weight was lower than expected and some extra signals observed in the ^1^H NMR suggested that the polymerization was not controlled and some side reactions were occurring (Supplementary Materials). When using 0.25 equiv. of TEA, although the polymerization was slower only the signals attributed to dopamine initiated PLLA were observed. The catalyst concentration was increased from 0.25 to 1.00 and we did not observe any significant change in the polymerization kinetics (Table 1, entry 2–5 and Figure S12). Therefore, the minimum amount of catalyst used to carry out the polymerization was 0.25.

**Figure 1:**
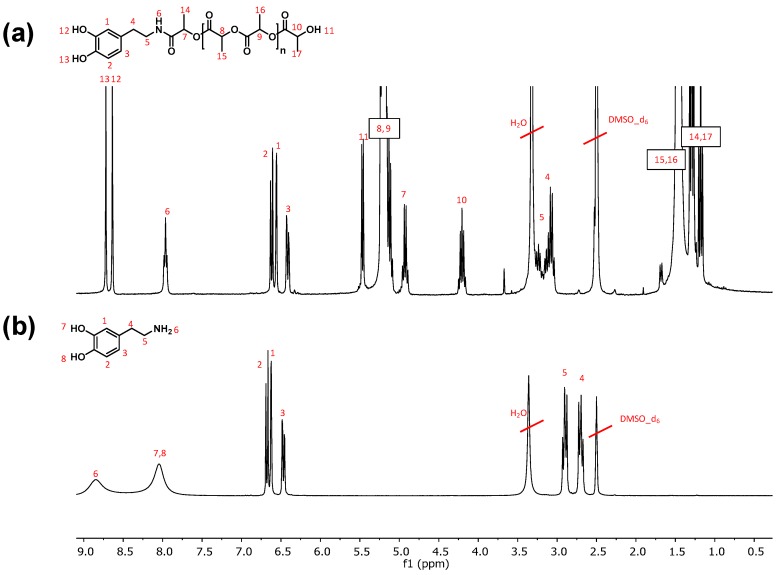
^1^H NMR spectra in DMSO-d_6_ for (**a**) Catechol end-functional PLLA with DP 10 and (**b**) Dopamine initiator.

Moreover, the diagnostic peak of dopamine units remain is the low-field shift of the CH_2_N signal (from δ 3.05 ppm in the amine to δ 3.25 ppm in the adduct). In addition to the characteristic signals associated with the CH_2_–NH and CH–OH moieties (at δ 3.25 and 4.21 ppm, with relative integrations of 2 and 1, respectively), a NHCO signal is observed at δ 7.96 ppm (relative integration 1), as seen in Figure 1a. The average *M*_n_ calculated by comparing the integration of protons from the catechol groups with that from the polymer backbones was 1700 g·mol^−1^, which was similar to the theoretical ones. The absence of side reactions was confirmed by carefully evaluating the carbonyl and the methine region in the ^13^C NMR were we did not observe any presence of racemization (Supplementary Materials Figure S3).

**Figure 2:**
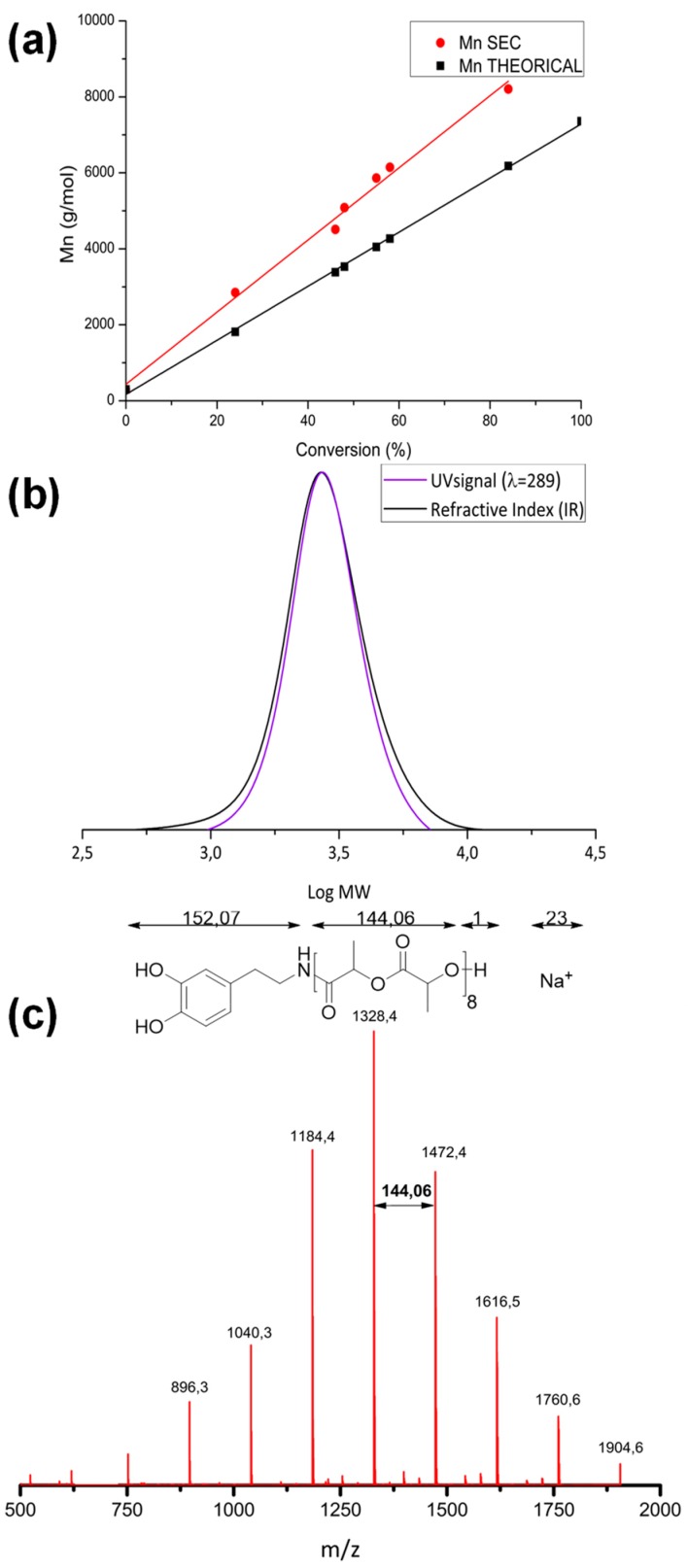
(**a**) Molecular weight vs. conversion graph of PLLA using dopamine as initiator, [(**a**) entry 7]; (**b**) SEC trace with UV (289 nm wavelength) and refractive-index signals for semitelechelic catechol PLLA; (**c**) MALDI-TOF spectra for semitelechelic catechol-PLLA. [(**b**,**c**) Entry 2].

As seen in Figure 2a, a linear relationship between molecular weight characterized by SEC and conversion was observed. In order to further confirm end-group fidelity and the absence of side reactions we analyzed the SEC traces using both ultraviolet/visible (UV−vis) and refractive index (RI) signals. As dopamine is UV active at 289 nm, all the polymer chains initiated with dopamine should overlap with the RI signal. As shown in Figure 2b, the UV−vis and RI SEC traces for PLLA overlay, indicating that dopamine is on the chain end, confirming that the catechol group is attached to the polymer chain. In order to further verify the polymerization, MALDI-TOF results were carried out (Figure 2c) where the corresponding end groups and only one set of peaks or distributions separated by 144 g·mol^−1^ (associated with the lactide monomer unit) were detected. Moreover, we investigated the potential of TEA to prepare higher-molecular-weight Dopamine-PLA. Therefore, the targeted degree of polymerization, [M]_0_/[I]_0_ was varied from 10 to 100 (Supplementary Materials Figure S4–S11). We found that the experimental molecular weight in both cases was similar to the targeted one (Table 1, entry 2 and 8) and still only the signals attributed to dopamine chain ends were observed in ^1^H NMR.

### 3.2. Characterization of the Redox Behaviour of Catechol-PLLA

**Figure 3:**
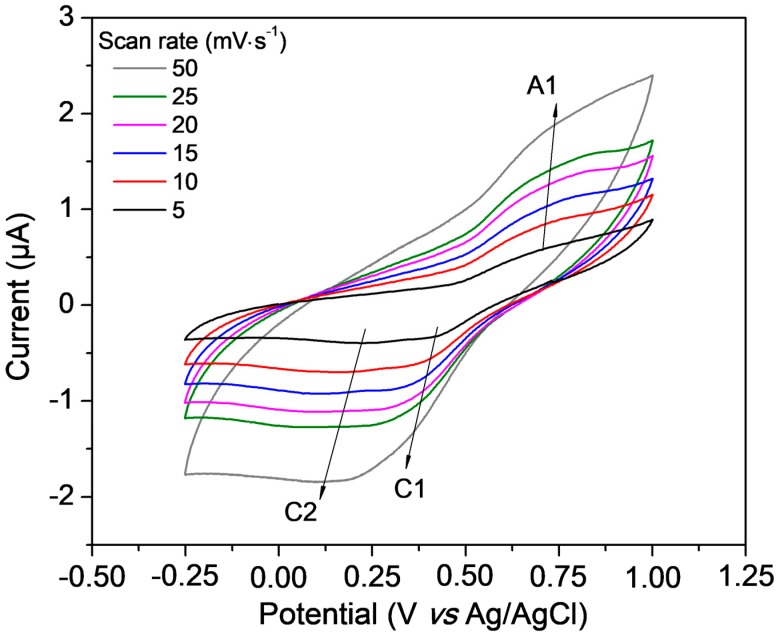
Cyclic voltammograms of catechol end-functional PLLA (drop-casted on a GC electrode) in 0.1 M HClO_4_ aqueous solution at various scan rates.

The redox behavior of catechol moiety is largely affected by the electrolyte and the presence of protons in the electrolyte solution [20]. Therefore, the redox behavior of Catechol-PLLA was investigated by cyclic voltammetry in acidic aqueous solutions. Figure 3, shows the cyclic voltammetry (CV) of Catechol-PLLA (drop-casted film in GC electrode) in 0.1 M HClO_4_ aqueous solution. One oxidation peak is observed in the anodic scan at 0.69 V (A1) and two reduction peaks at 0.43 V (C1) and 0.23 V (C2) at a 5 mV·s^−1^ scan rate. These redox peaks correspond to the oxidation and reduction processes from catechol to o-quinone via a two-electron two-proton process [16]. When increasing the scan rate, a slight shift is observed towards positive and negative potential in the anodic and cathodic peaks, respectively. The peak currents show a linear dependence on the square root of the scan rate indicating that the redox processes of PLLA-catechol are diffusion-controlled. This result confirms that the catechol moiety remained untouched during the polymerization of PLLA.

### 3.3. Post-Functionalization of Semitelechelic Catechol-PLLA

**Figure 4:**
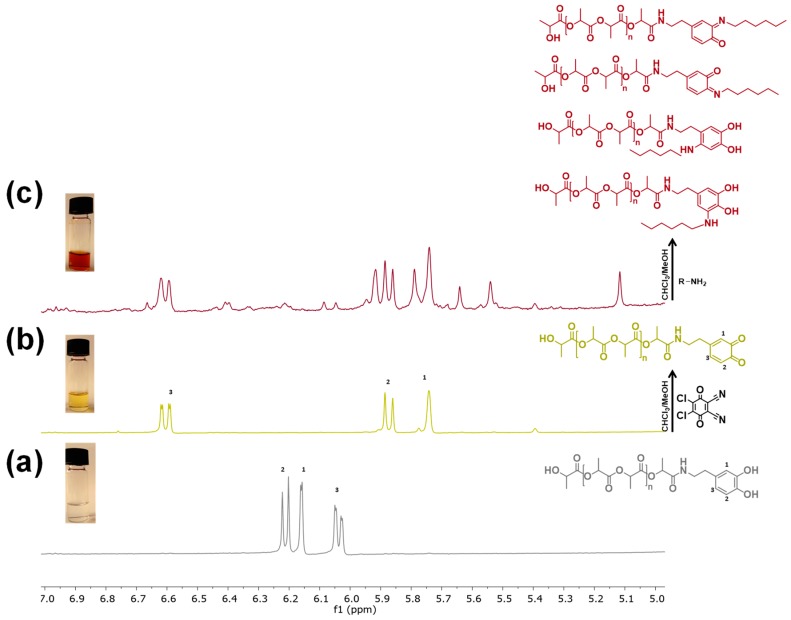
Scale spanded ^1^ H NMR of catechol-PLLA (**a**) before oxidation; (**b**) after oxidation and (**c**) after post-functionalizatoin with hexamethylenamine.

One of the advantages of catechol units is their ability to be easily oxidized into highly reactive o-quinones allowing its functionalization either by Michael addition or Schiff base reaction. The reaction between catechols and amines is vital in biological processes such as the crosslinking of adhesive proteins by marine organism [21]. In order to convert the catechol to ortho-quinone, the catechol was oxidized using 2,3-Dichloro-5,6-dicyano-1,4-benzoquinone (DDQ) (1 equiv.) in CDCl_3_/CH_3_OH solution. The solution changes color indicating that some reactions might have been taking place. To verify the quinone formation, we followed the change in the characteristic signals of dopamine aromatic region by ^1^H NMR spectroscopy. We found that due the higher electron-withdrawing property of the carbonyl groups there is an effect in the shielding effect on the protons confirming that the catechol was converted to ortho-quinone. Figure 4c shows the representative ^1^H NMR spectra of the reaction of oxidized catechol and hexylamine. As soon as the reagents were mixed, the solution turned dark red. The amine can react through two different mechanisms with the o-quinone via the Michael addition or via Schiff base reaction. When the Michael addition is carried out, the amine attach to the quinone in beta position, and via Schiff base an imine group is formed [9,22]. As expected hexamine has been introduced through two mechanisms, on the one hand the peaks corresponding to the Michael addition (5.1 and 5.5 ppm) are observed together with the peaks attributed to the formation of imine (5.7, 5.8 and 6.6 ppm).

### 3.4. Synthesis and Characterization of Silver Nanoparticles Using Catechol End-Capped PLLA Polymer

**Figure 5:**
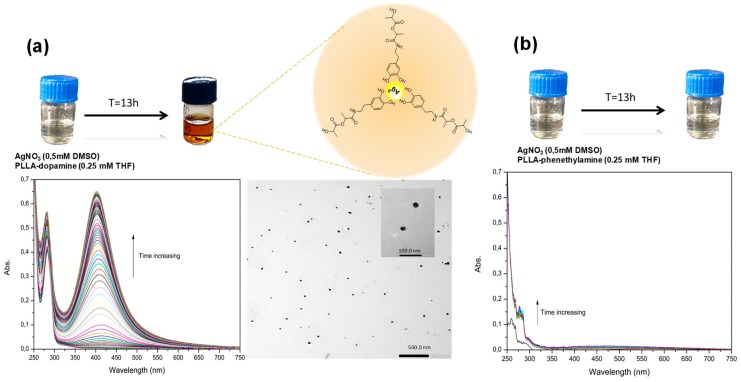
(**a**) The progress of the Ag^+^ reduction in the presence of catechol-PLLA studied by extinction spectroscopy and TEM image of AgNPs stabilized by the polymer; (**b**) The progress of the Ag^+^ reduction in the presence of PLLA-phenethylamine studied by extinction spectroscopy.

Catechol is also known to have the ability to reduce metal ions to metal nanoparticles. For instance, elemental AgNPs (Ag^0^) with small particle sizes were already prepared by adding a silver nitrate (AgNO_3_) aqueous solution to a catechol containing polymers [10]. Indeed, the catechol can reduce the Ag^+^ into Ag^0^ while the polymer can stabilize the so-formed nanoparticles. Therefore, we explored the utilization of catechol end-functional PLLA for the synthesis of AgNPs using the catechol group as reducing agent and the PLLA with a degree of polymerization of 50 polymer chain as steric stabilizer. For comparative purposes PLLA without catechol units was also investigated as precursor for the synthesis of AgNPs. In this regard, PLLA with a degree of polymerization of 50 was synthesized using phenethylamine as initiator (Table 1, entry 9). The formation of the silver NPs was monitored with respect to time by extinction spectroscopy. A single, narrow surface plasmon resonance band (SPRB) with a maximum at 402 nm and a full width at half maximum (FWHM) of 89 nm was obtained for the polymer-Ag system, which is indicative of the formation of non-aggregated, uniformly-sized nanoparticles (Figure 5a). A yellowish coloration of the dispersion as well as the transmission electron microscopy (TEM) images confirmed the synthesis of these AgNPs (Figure 5a). The nanoparticles size distribution was studied using the ImageJ software where particles smaller than 50 nm were observed (Figure 5a). In the control experiment without catechol units we did not observed any nanaopaticles formation (Figure 5b).

## 4. Conclusions

In this work, the controlled organocatalyzed ROP of l-lactide using dopamine as initiator is shown. It was demonstrated that the dopamine can be used as initiator for the ring-opening polymerization of cyclic monomers such as l-lactide in the presence of triethylamine as catalyst. The polymers were analyzed by ^1^H NMR, SEC-IR and MALDI-TOF and the results confirmed the controlled nature of the polymerization and end-group fidelity. We show that this catechol end-functional PLLA polymer can be easily converted to highly reactive ortho-quinone using 3-Dichloro-5,6-dicyano-1,4-benzoquinone oxidizer. After the oxidation step, we confirmed that the catechol-PLLA polymer can be post-functionalized with amines. Besides the ability of catechol to be post-functionalized, we also confirmed the ability of catechol functionalized PLLA to reduce metal ions to metal nanoparticles, studying the formation of silver nanoparticles. It is expected that this new route of preparing catechol-PLLA polymers without protection will increase the accessibility of catechol containing biodegradable polymers by ROP.

## Figures and Tables

**Table 1:** Different synthesis routes for the polymerization l-lactide initiated by dopamine.

Entry	Initiator	Time (h)	Conv. ^1^ (%)	([M]_0_/[I]_0_/[Cat])	*M*_n_ (Theo) ^2^ (kg/mol)	*M*_n_ (Exp) ^3^ (kg/mol)	*Ð* ^3^
1	Dopamine	96	1	10/1/0	0	-	-
2	Dopamine	24	93	10/1/0.25	1.5	3.8	1.2
3	Dopamine	24	93	10/1/0.5	1.5	3.5	1.1
4	Dopamine	12	94	10/1/0.75	1.5	3.4	1.2
5	Dopamine	12	94	10/1/1	1.5	3.6	1.1
6	Dopamine	24	93	20/1/0.25	2.8	4.0	1.2
7	Dopamine	48	92	50/1/0.25	6.9	9.5	1.2
8	Dopamine	48	91	100/1/0.25	13.4	21.0	1.4
9	Phenethylamine	24	90	10/1/0.25	1.5	1.6	1.2

Reaction conditions: 2 mol·L^−1^ solution of l-lactide in CHCl_3_ at 25 °C using TEA as catalyst. ^1^ Conversion by ^1^H NMR; ^2^ Calculated from the molar mass of l-lactide (144 g·mol^−1^) × conversion × [the initial monomer]/[initiator ratio] plus the molar mass of the initiator. ^3^ Molecular weight (*M*_n_) and dispersity (*Ð*) obtained from size exclusion chromatography analysis in tetrahydrofuran relative to polystyrene standards.

## Supplementary Materials

The following are available online at http://www.mdpi.com/2073-4360/10/2/155/s1, Table S1: Different synthesis rout for the polymerization l-lactide initiate by dopamine, Figure S1: Kinetics of polylactide followed by ^1^H NMR ( monomer 5.45 ppm polymer 5.20 ppm) (entry 2 of Table 1), Figure S2: ^1^H NMR of catechol-PLLA for degree of polymerization. DP = 10. Reaction conditions: 2 mol·L^−1^ solution of l-lactide in CHCl_3_ at 25 °C using DBU as catalyst (entry 9 of Table S1), Figure S3: ^13^C NMR of Catechol-PLLA for degree of polymerization DP = 10 (entry 2 of Table 1), Figure S4: ^1^H NMR of Catechol-PLLA for degree of polymerization DP = 20 (entry 6 of Table 1), Figure S5: SEC trace with UV (289 nm wavelength) and refractive-index signals for semitelechelic for DP = 20 (entry 6 of Table 1), Figure S6: MALDI-TOFF spectra for semitelechelic catechol PLLA of DP = 20 (entry 6 of Table 1), Figure S7: ^1^H NMR of Catechol-PLLA for degree of polymerization DP = 50 (entry 7 of Table 1), Figure S8: ^13^C NMR of Catechol-PLLA for degree of polymerization DP = 50 (entry 7 of Table 1), Figure S9: SEC trace with UV (289 nm wavelength) and refractive-index signals for semitelechelic for DP = 50 (entry 7 of Table 1), Figure S10: MALDI-TOFF spectra for semitelechelic catechol-PLLA of DP = 50 (entry 7 of Table 1), Figure S11: ^1^H NMR of Catechol-PLLA for degree of polymerization DP = 100 (entry 8 of Table 1), Figure S12: Kinetic plots for the different experiments runned with different TEA concentratios.
