# Supplementary material for: Catechol End-Functionalized Polylactide by Organocatalyzed Ring-Opening Polymerization
Source: Polymers (Basel). 2018 Feb 6;10(2):155. doi: 10.3390/polym10020155 (PMC6415175; doi:10.3390/polym10020155)
Supplement: Supplementary file 1 [file polymers-10-00155-s001.pdf]

Catechol end-functionalized Polylactide by organocatalyzed ring-opening polymerization

Naroa Sadaba[1,2], Maitane Salsamendi[1], Nerea Casado[1], Ester Zuza[2], Jone Munoz[2], Jose-Ramon Sarasua[2], David Mecerreyes[1,3], Daniele Mantione[1], Christophe Detrembleur[4], Haritz Sardon[1,3*]

[1] POLYMAT, University of the Basque Country UPV/EHU, Joxe Maria Korta Center, Avenida Tolosa 72, Donostia/SanSebastian 20018, Spain
[2] Department of Mining-Metallurgy Engineering and Materials Science, POLYMAT, University of the Basque Country UPV/EHU, School of Engineering, Alameda de Urquijo s/n, Bilbao 48013, Spain
[3] IKERBASQUE Basque Foundation for Science, Maria Diaz de Haro 3, E-48013 Bilbao, Spain
[4] Center for Education and Research on Macromolecules (CERM), CESAM Research Unit, University of Liège (ULg), Sart Tilman B6a, LIEGE, Belgium
* Correspondence: haritz.sardon@ehu.es; Tel.: +34-94-301-5303

Table S1: Different synthesis rout for the polymerization L-lactide initiate by dopamine.

	Solvent	Temperature (ºC)	CATALYST	Time(h)	[M]/[I]/[Cat]	DP_{NMR}
**1**	DCM	25	-	96	10/1/0	8
**2**	$CHCl_3$	25	-	96	10/1/0	12
**3**	DMF	25	-	48	10/1/0	9
**5**	THF	25	-	96	10/1/0	7
**6**	THF	50	-	48	10/1/0	7
**7**	DMF+$CHCl_3$	25	-	144	10/1/0	-
**8**	DMF+$CHCl_3$	25	TEA	24	10/1/1	11
**9**	DMF+$CHCl_3$	25	DBU	48	10/1/1	

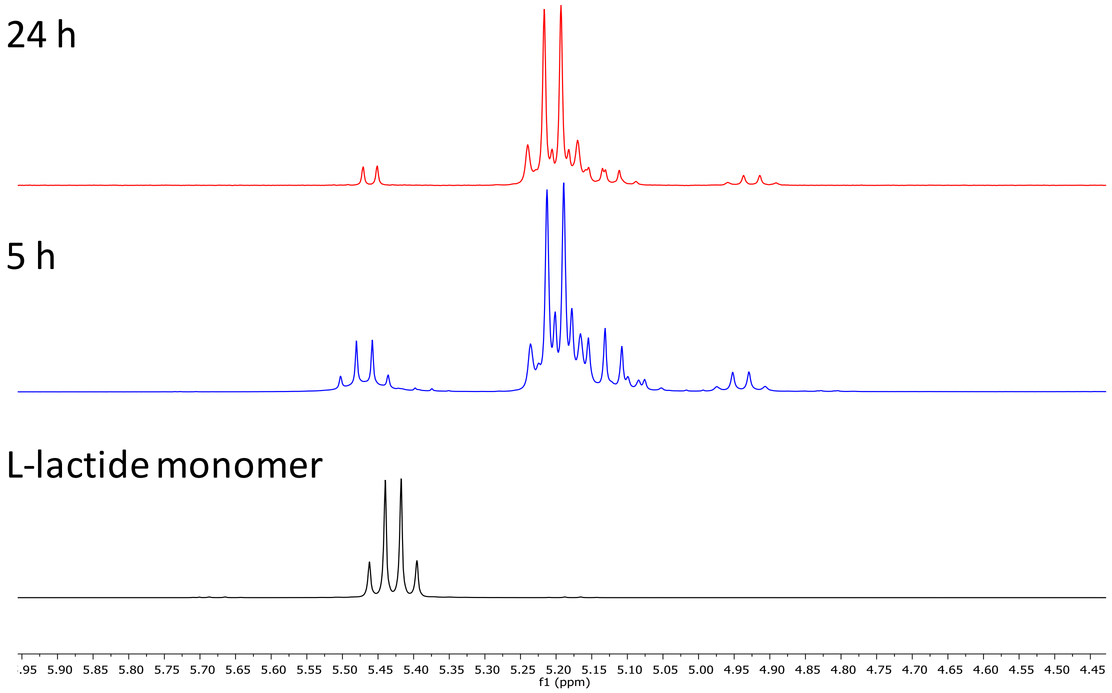

Figure S1: Kinetics of polylactide followed by ^{1}H NMR (monomer 5.45 ppm polymer 5.20 ppm) (entry 2 of table 1).

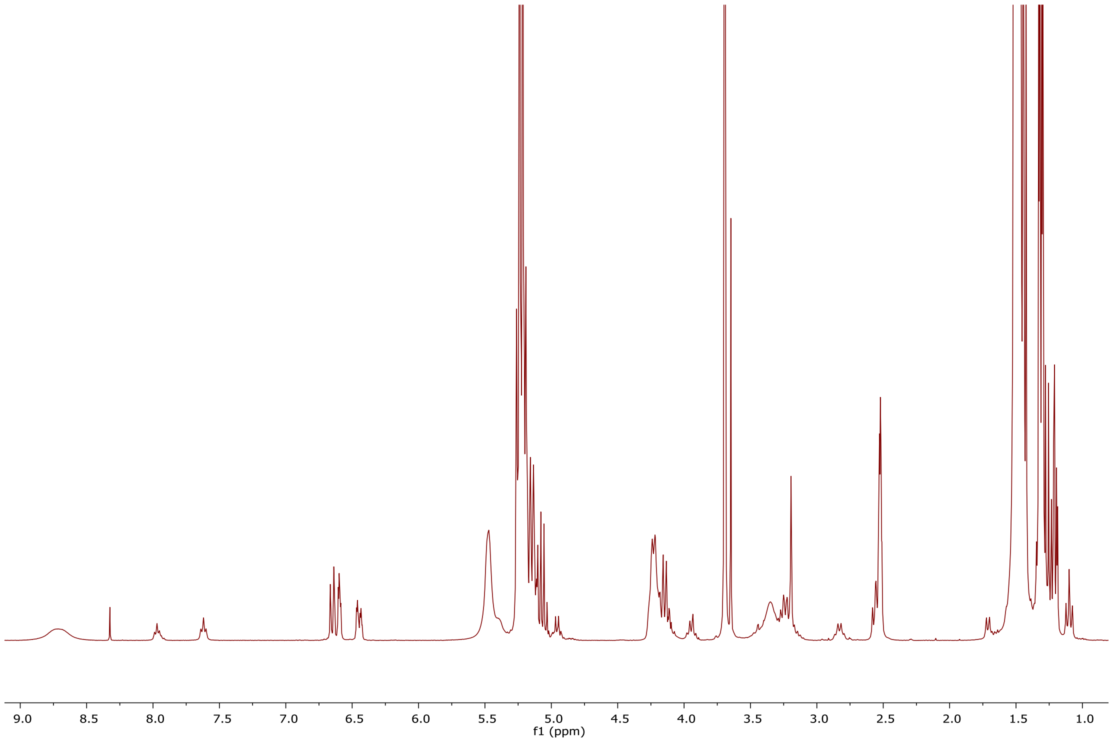

Figure S2: ^{1}H NMR of Catechol-PLLA for degree of polymerization. Dp = 10. Reaction conditions: 2 mol L^{-1} solution of L-lactide in $CHCl_3$ at 25 °C using DBU as catalyst. (entry 9 of table S1).

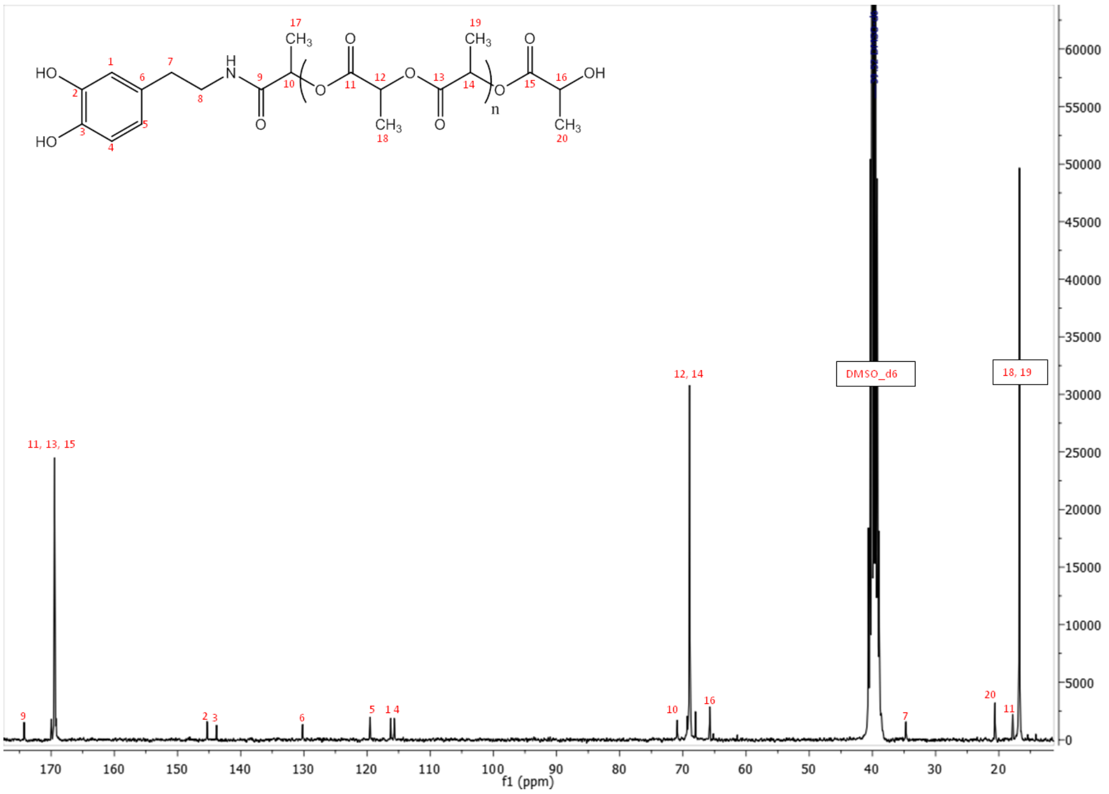

Figure S3:^{13}C NMR of Catechol-PLLA for degree of polymerization DP = 10. (entry 2 of table 1).

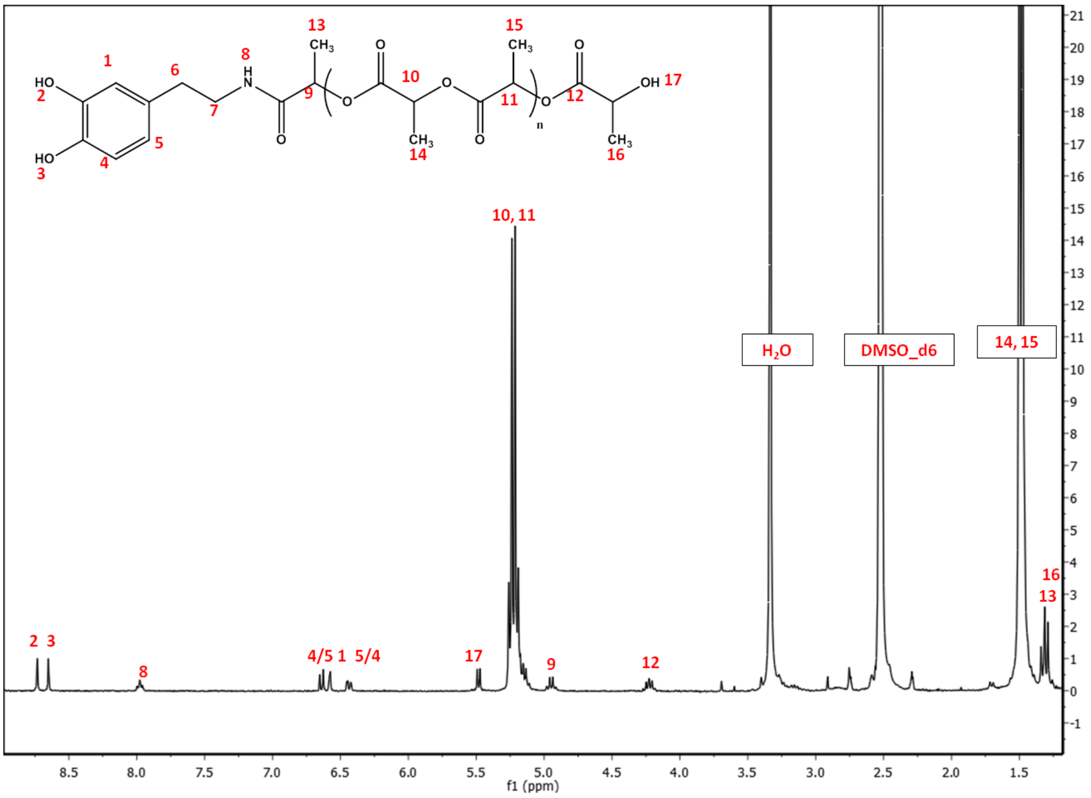

Figure S4: ^{1}H NMR of Catechol-PLLA for degree of polymerization DP = 20. (entry 6 of table 1).

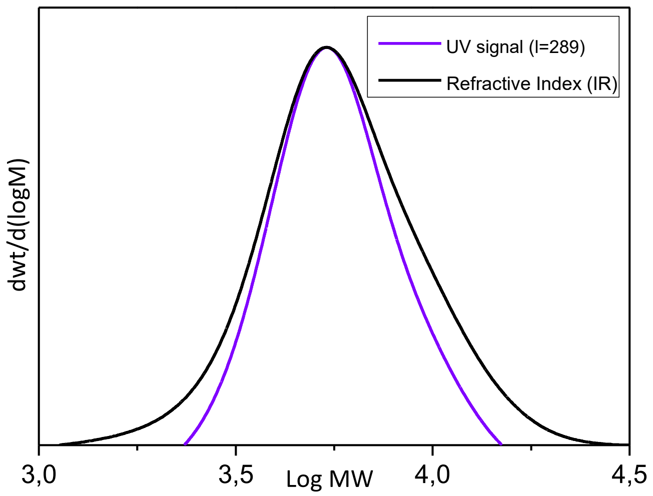

Figure S5: SEC trace with UV (289 nm wavelength) and refractive-index signals for semitelechelic for DP = 20. (entry 6 of table 1).

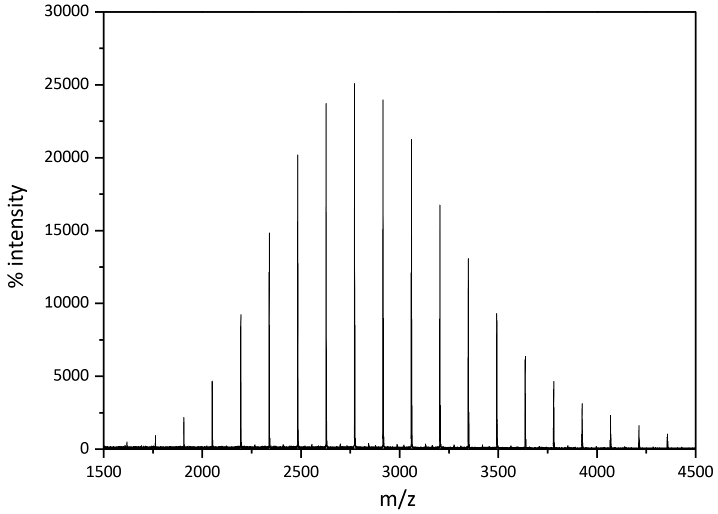

Figure S6: MALDI-TOFF spectra for semitelechelic catechol PLLA of DP = 20 (entry 6 of table 1).

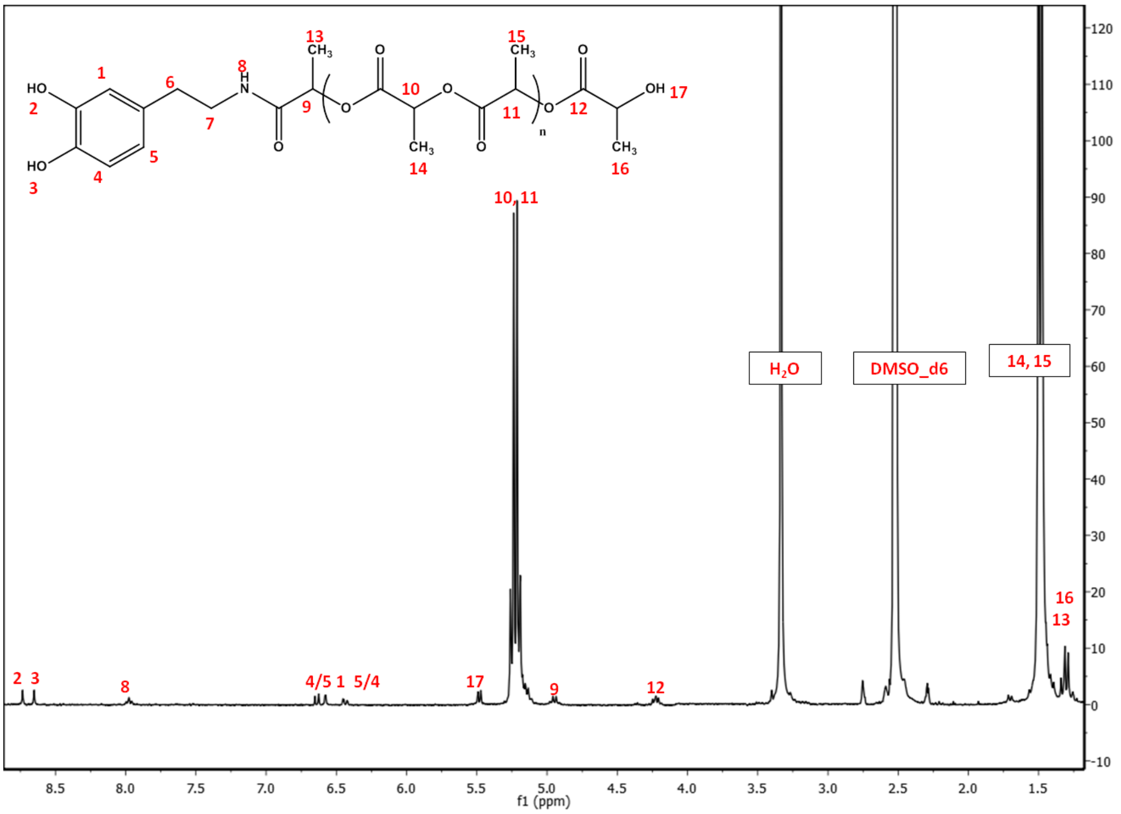

Figure S7: ^{1}H NMR of Catechol-PLLA for degree of polymerization DP = 50. (entry 7 of table 1).

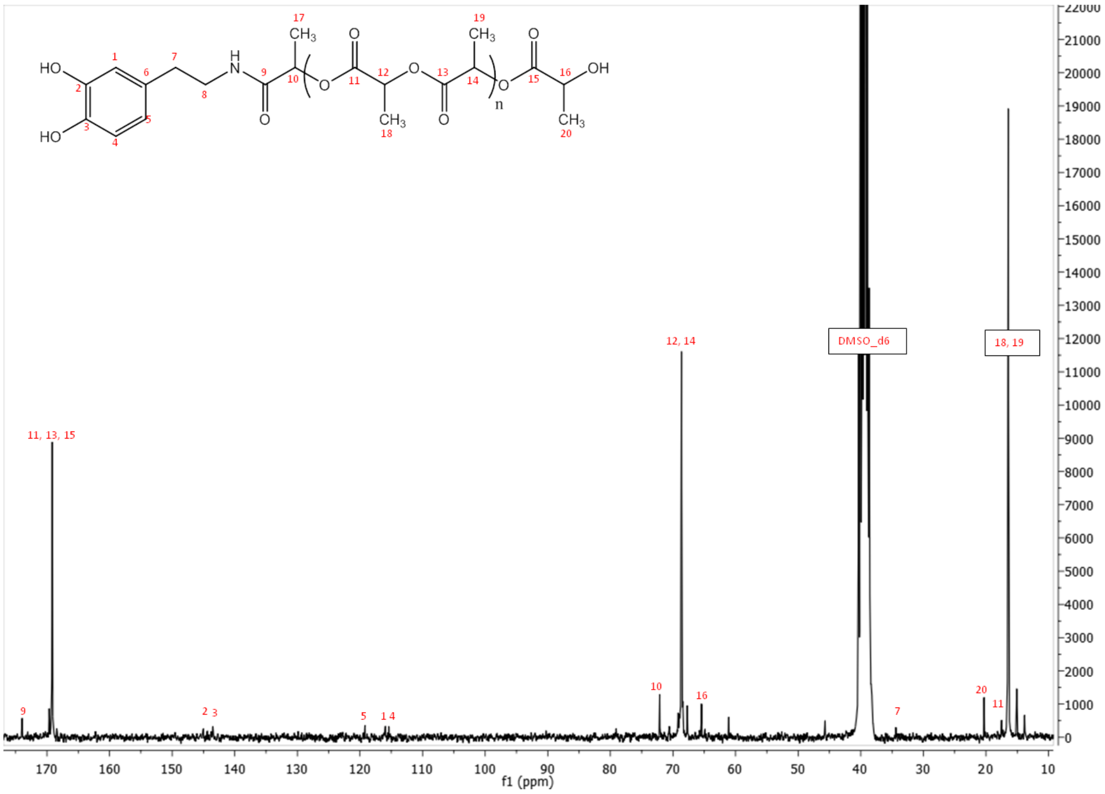

Figure S8: ^{13}C NMR of Catechol-PLLA for degree of polymerization DP = 50. (entry 7 of table 1).

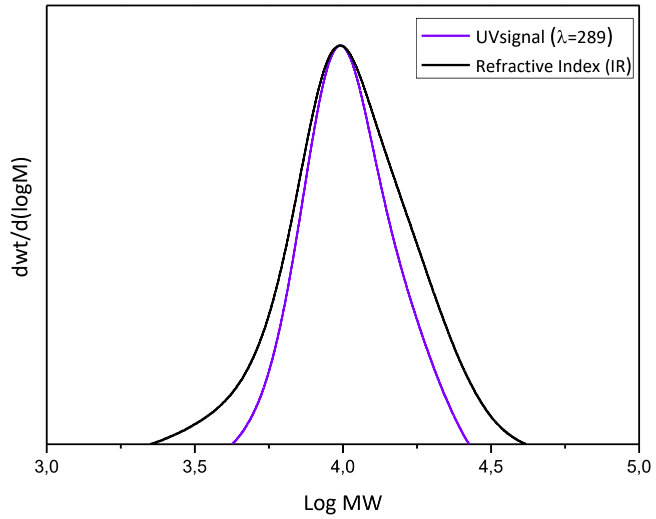

Figure S9: SEC trace with UV (289 nm wavelength) and refractive-index signals for semitelechelic for DP = 50. (entry 7 of table 1).

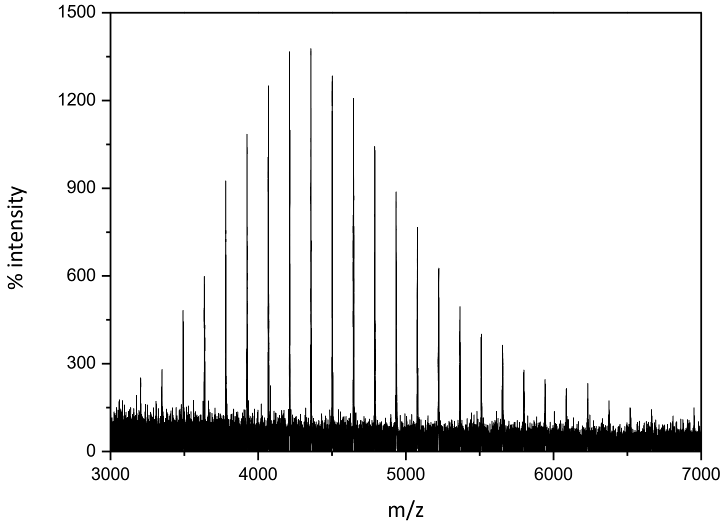

Figure S10: MALDI-TOFF spectra for semitelechelic catechol-PLLA of DP = 50 (entry 7 of table 1).

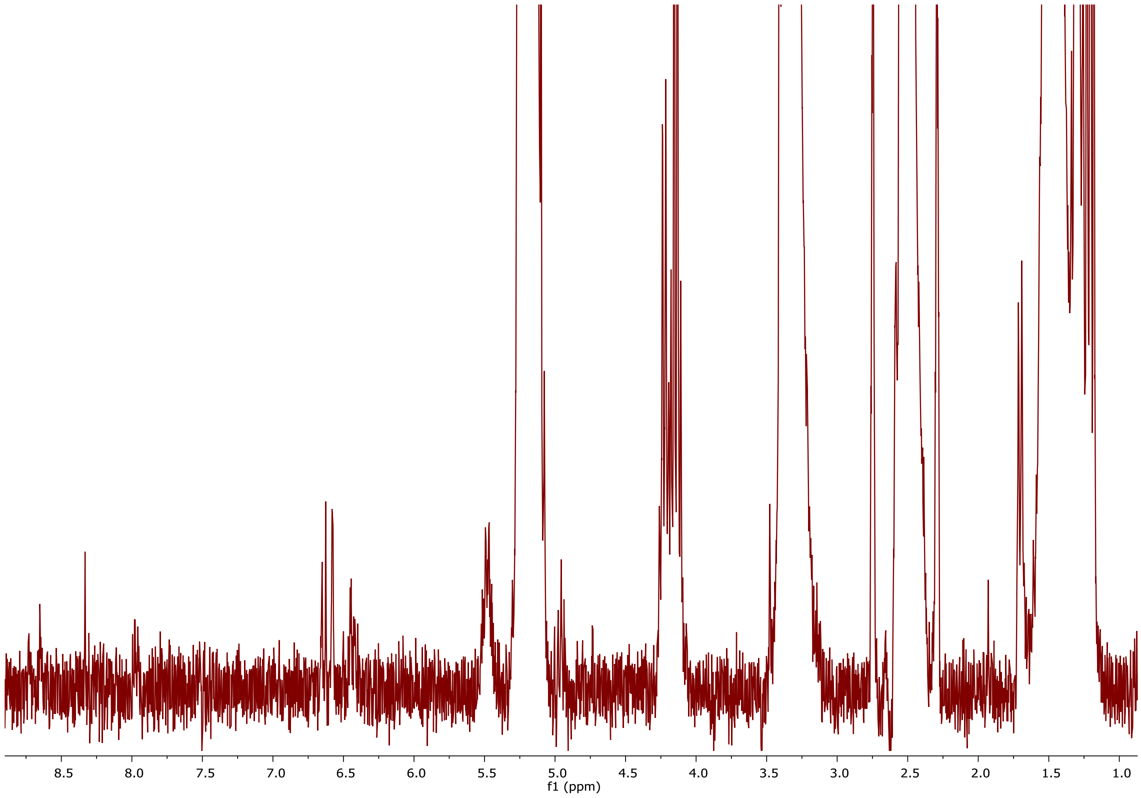

Figure S11: ^{1}H NMR of Catechol-PLLA for degree of polymerization DP = 100. (entry 8 of table 1).

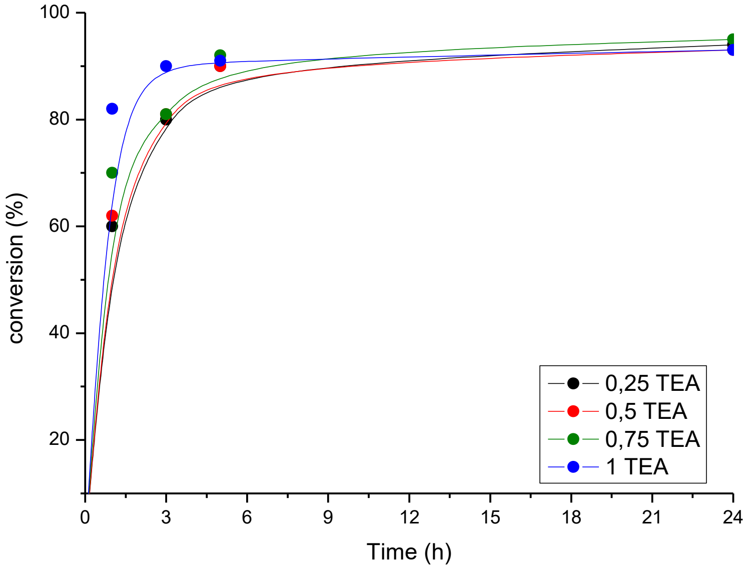

Figure S12: Kinetic plots for the different experiments runned with different TEA concentratios.
